# Implementation strategies in the context of medication reconciliation: a qualitative study

**DOI:** 10.1186/s43058-021-00162-5

**Published:** 2021-06-10

**Authors:** Deonni P. Stolldorf, Sheila H. Ridner, Timothy J. Vogus, Christianne L. Roumie, Jeffrey L. Schnipper, Mary S. Dietrich, David G. Schlundt, Sunil Kripalani

**Affiliations:** 1 Vanderbilt University School of Nursing, 461 21st Ave S., Nashville, TN USA; 2 Vanderbilt University Owen Graduate School of Management, 401 21st Ave S., Nashville, TN USA; 3 Vanderbilt University Medical Center, 1211 Medical Center Drive, Nashville, TN 37232 USA; 4 VA Tennessee Valley Healthcare System, 1310 24th Ave S., Nashville, TN 37212 USA; 5 Division of General Internal Medicine, Brigham and Women’s Hospital and Harvard Medical School, 1620 Tremont St., Boston, MA USA; 6 Vanderbilt University School of Medicine, Vanderbilt University School of Nursing, Nashville, TN USA; 7 Vanderbilt University Department of Psychology, 323 Wilson Hall, 2301 Vanderbilt Place, Nashville, TN 37240 USA; 8 Center for Clinical Quality and Implementation Research, Vanderbilt University Medical Center, 2525 West End Ave, Suite 1200, Nashville, TN 37203 USA

**Keywords:** Implementation strategies, ERIC, Medication reconciliation, Quality improvement

## Abstract

**Background:**

Medication reconciliation (MedRec) is an important patient safety initiative that aims to prevent patient harm from medication errors. Yet, the implementation and sustainability of MedRec interventions have been challenging due to contextual barriers like the lack of interprofessional communication (among pharmacists, nurses, and providers) and limited organizational capacity. How to best implement MedRec interventions remains unclear. Guided by the Expert Recommendations for Implementing Change (ERIC) taxonomy, we report the differing strategies hospital implementation teams used to implement an evidence-based MedRec Toolkit (the MARQUIS Toolkit).

**Methods:**

A qualitative study was conducted with implementation teams and executive leaders of hospitals participating in the federally funded “Implementation of a Medication Reconciliation Toolkit to Improve Patient Safety” (known as MARQUIS2) research study. Data consisted of transcripts from web-based focus groups and individual interviews, as well as meeting minutes. Interview data were transcribed and analyzed using content analysis and the constant comparison technique.

**Results:**

Data were collected from 16 hospitals using 2 focus groups, 3 group interviews, and 11 individual interviews, 10 sites’ meeting minutes, and an email interview of an executive. Major categories of implementation strategies predominantly mirrored the ERIC strategies of “Plan,” “Educate,” “Restructure,” and “Quality Management.” Participants rarely used the ERIC strategies of finance and attending to policy context. Two new non-ERIC categories of strategies emerged—“Integration” and “Professional roles and responsibilities.” Of the 73 specific strategies in the ERIC taxonomy, 32 were used to implement the MARQUIS Toolkit and 11 new, and non-ERIC strategies were identified (e.g., aligning with existing initiatives and professional roles and responsibilities).

**Conclusions:**

Complex interventions like the MARQUIS MedRec Toolkit can benefit from the ERIC taxonomy, but adaptations and new strategies (and even categories) are necessary to fully capture the range of approaches to implementation.

**Supplementary Information:**

The online version contains supplementary material available at 10.1186/s43058-021-00162-5.

Contribution to the literature

- Identifies the strategies most commonly used in implementing medication reconciliation (MedRec) interventions, an important patient safety initiative.
- Applies the Expert Recommendations for Implementing Change (ERIC) taxonomy to identify mechanisms teams used to implement a complex and consequential healthcare intervention like MedRec.
- Demonstrates the value of the ERIC taxonomy for guiding implementation as well as gaps by identifying two additional categories and expanding the taxonomy.
- Identifies specific mechanism implementation teams used to execute implementation strategies.

## Background

More than 50% of hospitalized patients experience one or more unintentional medication discrepancies (UMDs), defined as unexplained differences in medication regimens across different sites of care, with some having the potential for significant harm [1–8]. The majority occur during care transitions, when patients are admitted, transferred between units, or discharged home [9]. Medication reconciliation (MedRec) reduces the risk of UMDs and harmful adverse drug events [6, 7, 10–13] that increase hospitalization cost and length of stay [10, 14]. MedRec is the process of “creating the most accurate list possible of all medications a patient is taking and comparing that list against the physician’s admission, transfer, and/or discharge orders, with the goal of providing correct medications to the patient at all transition points within the hospital” [15].

MedRec implementation is complex. It requires extensive use of resources [8, 16], wide-scale organizational change [17–19], interprofessional collaboration [20], and workflow redesign [8, 16, 19, 21, 22]. Reports demonstrate these complexities create multiple barriers to successful MedRec implementation including a lack of role clarity, insufficient interprofessional collaboration, and limited organizational capacity including personnel resources and time commitments [10, 19, 22–25]. Despite identified MedRec implementation challenges [19, 20], and growing recognition that implementation strategies are key to successfully adopting and implementing healthcare interventions, existing MedRec studies focus predominantly on clinical outcomes of MedRec [26–29]. We know comparatively little about the specific strategies used to effectively implement MedRec interventions [30]. Furthermore, MedRec provides a good context to assess how well an existing taxonomy can be applied to describe implementation strategies used for this complex healthcare intervention. Careful consideration of *how* MedRec interventions are implemented and deployed in hospitals may help future efforts to overcome initial implementation barriers to successfully adopt MedRec and ensure their long-term sustainability.

### Implementation strategies

Implementation strategies, defined as systematic processes used to adopt and integrate evidence-based healthcare interventions into usual care within specific settings [31], can be discrete (e.g., education) or multifaceted (e.g., asses for readiness and identify barriers) and have varying degrees of complexity (e.g., reallocate staff and change workflow). The Expert Recommendations for Implementing Change (ERIC) taxonomy [30] summarizes 73 discrete implementation strategies into six categories including plan (e.g., conduct a local need assessment), educate (e.g., develop educational materials), finance (e.g., fund clinical innovation), restructure (e.g., change record systems), quality management (e.g., audit and feedback systems), and attend to policy context (e.g., change accreditation or membership requirements). Despite general guidance regarding the effective deployment of implementation strategies [32], studies of MedRec have yet to offer detailed descriptions of the specific strategies, their deployment, and effectiveness.

### MARQUIS and MARQUIS2

The Multicenter Medication Reconciliation Quality Improvement Study (i.e., MARQUIS) [1, 33] and the Implementation of a Medication Reconciliation Toolkit to Improve Patient Safety study (i.e., MARQUIS2) [25, 33], developed an evidence-based MedRec Toolkit (i.e., MARQUIS Toolkit) and evaluated the effect of the toolkit on medication discrepancies in 5 hospitals and 18 hospitals, respectively. Seventeen system-level interventions representing 8 domains comprise the latest version of the MARQUIS Toolkit [33]. In MARQUIS2, the setting for this current analysis, sites worked with an experienced mentor to implement the toolkit, with each site implementing 1 to 15 toolkit components. The most common were taking a best possible medication history (BPMH), identifying high-risk patients, clarifying roles and responsibilities among clinical staff, providing audit and feedback on MedRec roles, and identifying high-risk patients. A mentor, who had expertise in MedRec and quality improvement, was assigned to each site and conducted one site visit and monthly mentor meetings to help guide implementation. However, mentors provided only general implementation guidance (e.g., identify key stakeholders, assemble a multidisciplinary QI team analyze care delivery, and track performance)). Thus, implementation of the MARQUIS Toolkit depended upon the unique local conditions facing mentors and local implementation teams.

### Objective

The first author leveraged the MARQUIS2 study to investigate contextual factors as potential drivers in the selection of implementation strategies and subsequent sustainability. As part of that study, the authors conducted a robust qualitative exploration of strategies hospitals used to implement the toolkit. This paper reports MARQUIS2 Toolkit implementation strategies and how implementation teams operationalized these strategies. Understanding these strategies and their associated operationalizations are important as MARQUIS is recognized as the premier evidence-based approach to MedRec and is being spread through the Society of Hospital Medicine’s national collaborative [34].

## Methods

### Study design and sample

We designed a cross-sectional descriptive qualitative study using content analysis and the constant comparison technique. Using purposive sampling, the principal investigator (PI) (DPS) recruited implementation teams, site leaders, and executive leaders for interviews. Recruitment strategies included emails and/or phone calls, phone/videoconferencing, a study flyer, and participation in monthly mentor meetings. The PI worked closely with the MARQUIS2 research team and project staff to facilitate recruitment, including obtaining a contact list of site leaders and team members from project staff and MARQUIS research staff introducing the PI and her research study to the 18 sites during mentor meetings. Site leaders aided in recruitment efforts and gaining access to eligible executive leaders. A recruitment letter was emailed to all potential participants, followed by two reminders sent at 7 and 14 days from the initial solicitation. In some instances, site leaders preferred to keep contact information confidential and instead forward the study information to executive leaders. Eligible participants were (a) employees of hospitals that participated in MARQUIS2, (b) implementation team members, or (c) be executive leaders to whom the implementation team reported [e.g., Chief Medical Officer or Chief Nursing Officer].

### Data collection

The PI conduced web-based focus group (FGs) or interviews using Blue Jeans Network***®*** web conferencing. When fewer than four participants per hospital were recruited or team members were hesitant to participate in FGs, the PI conducted individual interviews. Using private office space and assigning pseudonyms for hospitals and participants ensured confidentiality. A semi-structured interview guide developed by the research team and consisting of main questions and prompts provided the structure for the 45–50-min interviews (average of 44 min). Select constructs from the Consolidated Framework for Implementation Research (CFIR) [35] and the Stages of Implementation Completion (SIC) framework [36] HA-guided question development and capturing three distinct phases (i.e., pre-implementation, implementation, and post-implementation). Interviews/FGs were digitally recorded and transcribed. Field notes helped to inform data analysis. After encountering recruitment difficulties, monthly mentor-call meeting minutes were collected and, as an alternative to personal interviews, executive leaders were asked to respond by email to five key interview guide questions identified by the research team. Even after data saturation was evident by repetition of themes and strategies, the PI completed additional interviews with all who agreed to be interviewed to obtain representation from as many MARQUIS2 hospitals as possible.

### Data analysis

The Miles and Huberman [37] framework of data reduction, data display, and drawing conclusions guided data analysis of interviews, meeting minutes, and email responses. Data reduction occurred through content analysis of transcribed interviews and focus groups, meeting minutes, and email responses. Codes were assigned to relevant data segments using an a priori codebook consisting of the six ERIC categories of plan, educate, finance, restructuring, quality management, and attend to policy context. New codes were assigned when needed and added to the Codebook. A research assistant and the PI independently coded the data and met to review codes and to resolve coding differences. The PI also consulted with faculty experts in qualitative research methods for guidance and trouble-shooting and used their Microsoft Excel template for coding the data. The accuracy of the data was facilitated through verbatim transcription and verifying the accuracy of transcription, the use of two coders and a codebook with clearly defined codes, and establishing an audit trail to record decision-making throughout the research process. The local university’s Institutional Review Board reviewed and approved the study. Informed consent was obtained prior to each focus group or interview.

## Results

### Sample characteristics

We invited 108 implementation team members and site leaders and 30 responded to our interview invitation (30% response rate). Of these, two declined to participate, one withdrew, and one respondent was unresponsive to emails to schedule the interview. Recruitment of hospital executive leaders was difficult as the researchers were dependent on-site contacts to facilitate access to leadership. Of those leaders who were contacted (*n* = 6), only two agreed to participate (33% response rate). To glean more representation, we offered an option for leaders to respond to five key interview questions via email and requested access to the MARQUIS2 monthly meeting minutes. The final sample consisted of 27 participants representing 14 sites, and we successfully conducted two focus groups (4–5 members/group), three group interviews (2–3 members/group), 11 individual interviews, and one email “interview.” Table 1 indicates participant characteristics. Ten sites agreed to share their minutes. With these additional data collection approaches, we were able to obtain data from 16 of the 18 sites. In the next section, major and minor themes and associated implementation strategies are reported and conclude with recommendations for others who wishes to implement the MARQUIS Toolkit.

**Table 1:** Participant characteristics

Profession	Nursing N (%)	Pharmacist N (%)	Physician N (%)	QI N (%)	Total N (%)
**Role in implementation**					
Implementation teams	0 (0)	13 (92.9)	0 (0)	1(7.1)	14(52)
Site leaders	0 (0)	5 (45.5)	5(45.5)	1 (9.1)	11(41)
Executives	1 (50)		1(50)		2(7)
**Total**	**1(3.6)**	**18(64.3)**	**7(25)**	**2(7.1)**	**27 (100)**

### Major themes of strategies important for MedRec implementation

Participants repeatedly reported the use of various implementation strategies, represented by the ERIC taxonomy, which were therefore classified as major themes. The overarching implementation themes, their theoretical definitions, and associated list of implementation strategies are listed in Table 2. Of the themes identified, restructuring, quality management, planning, and educating were the most frequently mentioned by participants. Within these themes, some strategies were also mentioned more often than others and these are noted in the various sections below.

**Table 2:** Implementation categories, associated strategies, and examples of operationalization

**Restructuring** **Strategies that altered staffing, professional roles, physical structures, equipment, and data systems to successfully implement the MARQUIS Toolkit**	
**ERIC implementation strategies**	**Examples of strategy operationalization (tactics)**
Revise professional roles	Included in the role of non-physician professionals the ability to document medication-related changes in the EHR. Pharmacy technicians documented in the EHR and took a BPMH. Dedicated pharmacist time to do MedRec instead of nurses and physicians. Decentralized pharmacy services. Moved the responsibility for training pharmacy technicians from physicians to pharmacists and pharmacy technicians. Used nurse practitioners in certain areas to correct medication lists.
Change records systems	Adopted electronic processes to support MedRec such as tracking boards and use of icons; processes to show flow and completion of MedRec stages; keyboard shortcuts to facilitate documentation. Transitioned from paper process to electronic processes to support MedRec. Adapted EHR systems to accommodate documentation of MedRec in the EHR. Created a progress note template in the EHR. Adapted EHR to be clear to show which patients had a BPMH done and who still required one. Used bed boards to identify patients who might still need medication history (high-risk patients).
**Other non-ERIC strategies in this category**	
Facilitate relay of clinical data to inter-professional teams	Used the EHR and existing technology to aid inter-professional information exchange such as using keyboard shortcuts for common phrases related to MedRec and icons on tracking boards. Used the EHR to share when pharmacist or pharmacy technician has reviewed medication lists. Standardized documentation in one repository to communicate the admission list was completed. Used progress notes in the EHR to relay information.
Change workflow systems	Restructured work or responsibilities to allow pharmacists dedicated time for MedRec. Adapted existing technology such as adding progress notes or board systems to follow patient flow. Used algorithms to flag high priority patients for pharmacy technicians to see. Connected to external pharmacy, care centers, and physician office EHRs for patient medication list. Integrated discharge orders with standardized and pre-selected boxes.
**Quality management** **Strategies that supported MARQUIS Toolkit implementation and evaluation of quality of care and fidelity to the toolkit. Examples included the use of data systems and support networks to collect data for monitoring and evaluation.**	
**ERIC implementation Strategies**	**Examples of strategy operationalization (tactics)**
Use advisory boards and workgroups	Determined board membership; Built a multi-professional team and focused on including professions “touched” by the improvement activities (e.g., clinical, operational, patient experience, physicians). Advisory board provided support to hospitals’ participation in collaborative.
Audit and provide feedback	Audited various aspects of MedRec: specific processes such as BPMH appropriateness and accuracy of medication lists; individuals doing MedRec; physician’s completeness of medication information. Observed staff conducting MedRec to evaluate, monitor, and provide feedback. Conducted daily discussions and use of data.
Purposefully re-examine implementation	Examined new structures (e.g., documentation and notes in EHR) with involvement of frontline staff and determine best practices. Re-evaluated best ways to use pharmacy technicians.
Use a quality improvement/implementation advisor	Identified and used a mentor internal to organization and include QI department staff or process improvement person from the beginning of the project. Made use of external mentors for discussions about challenges like time and resource limitations and for troubleshooting.
Develop tools for quality monitoring	Used Excel spreadsheet to track number of patients seen each day by pharmacy technicians. Built a note template into EHR with a screening element to monitor type and time used for doing interventions. Modified medication list status choices in EHR to reflect the workflow (pharmacy team initiated or in process, pharmacy technician history completed but pending pharmacist review, pharmacist review completed)
Obtain and use patient/consumer and family feedback	Shared ideas and findings, and changes staff wanted implemented. Provided feedback of efficacy of MedRec notes to ensure physicians used correct processes as desired by pharmacists. Improved EHR processes by using MD feedback. Used staff (pharmacy services, nursing, hospitalist, physician) feedback on what should be improved and how.
**Other non-ERIC strategies in this category**	
Determine project-related goals for individual performance measures	Created a scorecard of activities that pharmacy technicians completed. Monitored the number of medication histories completed within a week. Recognized units when 100% of their patients have completed MedRec. Monitored individual providers’ discrepancy rates. Expected pharmacy technicians to use two-source verification when conducting medication history.
Determine ownership and hold individuals accountable.	Assigned ownership of the project and held people accountable for their performance.
**Planning** Strategies used by hospitals to prepare their organization for participation in a multi-site quality improvement initiative called MARQUIS2. Plan strategies can help stakeholders gather data, select strategies, build buy-in, initiate leadership, develop relationships	
**ERIC implementation strategies**	**Examples of strategy operationalization (tactics)**
Gathering information*	
Conduct a local needs assessment	Used QI processes and surveys to identify areas for improvement and resources available. Used Gantt charts, swim lanes, and gap analysis to identify areas of need. Identified all requests for MedRec and where the biggest needs for MedRec were. Identified resource needs to justify resource requests. Information from needs assessment would drive the impact of the pharmacy team doing Med histories.
Assess for readiness and identify barriers	Determined project staffing, map existing processes and identify gaps that need addressing. Called meetings with frontline staff and organizational leaders to identify barriers. Implementation team represented all disciplines involved in MedRec (physicians, pharm tech, ED, pharmacists) to understand existing processes.
Identify resources	Specified who would be doing the work and collecting the data on these resources. Leveraged and used the large pharmacy residency program at the hospital to assist with the project. Identified staff that could be reallocated to meet project demands (e.g., nurse on light duty was trained and used to perform MedRec tasks)
Stakeholder buy-in*	
Identify and prepare champions	Delineated job functions/ Expectations of persons serving as champion. Identified those who were passionate about MedRec and recruited them to serve as champions. Some champions self-selected because they were motivated and identified MedRec improvement as an issue.
Involve executive boards and/or sponsors	Used quality improvement board, quality improvement section of the pharmacy department, and chief of quality improvement committee to promote and support the program and to make staff available and set structures in place for data collection. Executives promoted the program and encouraged applying to MARQUIS2; Obtained executives ‘permission to apply to MARQUIS program and to obtain resources to support implementation efforts. Garnered executives’ support for the project by having them help with selection of units for participation and set program expectations.
Recruit, designate, and train staff	Hired staff and trained new pharmacists. Used observation to provide training on how to perform medication histories Received hands-on training from another pharmacist. Identified individuals on existing steering committees to lead the MARQUIS project.
Conduct local consensus discussions	Set goals, expectations, and priorities during discussions. Conducted formal meetings with discussions with pharmacists and nursing staff on how to make changes to the MedRec process. Implementation teams held informal meetings and discussions with frontline pharmacists and pharmacy technicians to evaluate process and status of project. Conducted formal meetings with stakeholders to discuss how processes could improve when implementation problems occurred.
Marketing to stakeholders	Provided a bi-weekly tip sheets to physicians to raise awareness. Conducted noon lectures to house staff.
Select an implementation strategy*	
Tailor strategies to overcome barriers and honor preferences	Identified and discussed barriers, then strategies identified to overcome the barriers. Redesigned forms, obtained frontline staff feedback and made changes, with group consensus, to forms based on feedback.
Stage implementation scale up	Scaled up MedRec from when pharmacy technicians were available to also doing when they were off duty (nights/weekends). Achieved this by doing MedRec the next day when pharmacy technicians were available on those patients who were admitted overnight. Conducted a pilot on three units before scaling it up to other units.
Develop relationships*	
Build a coalition	Developed a MedRec committee and a team of hospitalists to work closely with the implementation team and site leader. Established a MedRec steering committee (MD lead, a MedRec pharmacist, regional pharmacy director, IT reps, QI specialist). Partnership with MARQUIS2 provided a structure for MedRec processes and specific project goals.
Partner with quality improvement experts external to the organization	Used QI staff to gather information, streamline processes, and identify high yield efforts.
**Other non-ERIC strategies in this category**	
Develop interdisciplinary implementation teams	Interdisciplinary teams used for implementation, including clinical coordinator, director of pharmacy, pharmacy residents, hospitalists. Teams consisted of pharmacy director, pharmacy manager, pharmacy coordinator, medical director.
Identify people and time necessary for data collection to support the project.	Determined persons responsible for data collection to support the project and identify resources needed to support data collection activities. Developed specific tools for data collection, interpretation and sharing - custom Excel spreadsheet, dashboard to assess productivity, repurposing existing reports to track pharmacist performance.
**Educate** **Strategies of various levels of intensity used to inform a range of stakeholders about the MARQUIS Toolkit and implementation efforts**	
**ERIC implementation strategies**	**Examples of strategy operationalization (tactics)**
Develop materials*	
Develop effective educational materials	Used electronic modules to broaden reach to staff Survey staff to identify learning needs. Created a “help” sheet for all the nursing units, inpatient-pharmacy, and hospitalists.
Educate*	
Provide ongoing consultation	Pharmacists were available to answer questions and to consult and troubleshoot with hospitalists and review with them quality reports. Implementation teams consulted with QI experts to streamline processes and address challenges to change behavior.
Conduct educational meetings	Used existing clinical (e.g., hospitalist meetings) and business meetings (e.g., quality improvement meetings) to provide education. Used different modalities including the intranet, in person feedback in clinical units, use of data, and role-play.
Make training dynamic	Used staff training in combination with shadowing pharmacy staff to verify accuracy of project-related processes; provided various modalities (pocket cards, online classes, and posters) available for self-paced learning.
Conduct educational outreach visits	Visited other successful MARQUIS sites or have them visit the hospital to provide training to project staff. Participated in a learning collaborative organized by MARQUIS project staff.
Conduct ongoing training	Conducted ongoing training through weekly emails and in person when needs presented.
Distribute educational materials	Communication occurred through distribution of educational materials that included using the Internet, creating help sheets, and using project generated toolkits and pocket cards.
Educate through peers*	
Inform local opinion leaders	Provided key stakeholders (like unit managers, service line directors) with information about the project (e.g., PowerPoint presentation to leadership), outcomes data, and project progress
Create or participate in a learning collaborative	Hospitals participated in a MARQUIS collaborative where sites presented their results and shared best practices with each other (online collaborative) and local one day workshops, where site leaders from several sites received in-person training. It also provided a chance for sites to meet leaders from other sites and to share their experiences.
Inform and influence stakeholders*	
Use mass media	Use TV advertisements, newspaper articles, education in the community, and marketing using social media. Used posters to inform hospital staff.
**Other non-ERIC strategies in this category**	
Individualized training sessions	Individual training sessions allowed for discussion of questions, answers, and immediate feedback; communicated performance on an individual basis; live training to allow trainees to ask questions. Train pharmacy technicians, shadowed them to ensure they were asking the right questions and putting the correct data into the charts, allowing them to work independently, and checking up to 20 records they completed to ensure history done correctly.
**Finance**	**Various finance strategies leveraged to incentivize the use of the MARQUIS Toolkit and provided resources for training and ongoing support.**
**Other non-ERIC strategies in this category**	
Incentivize positive performance and training.	Incentivized engagement with project using gift cards and other rewards.
Demonstrate value to justify program and gain ongoing support.	Used data to show the value of the program and its impacts on patients. Used pilot study to demonstrate return on investment.
**Policy context**	**Strategies that encouraged the adoption of the MARQUIS Toolkit through accrediting bodies, licensing boards, and legal systems.**
**ERIC implementation strategies**	**Examples of strategy operationalization (tactics)**
Use accreditation bodies and organizational policies to direct change.	Accreditation bodies required MedRec implementation and helped to enhance adoption/implementation. Organizational policies changed to allow lower level providers to correct the medication list to enhance the workflow.
**Integration (non-ERIC category)**	**Strategies that facilitated the integration of the new intervention(innovation) into existing structures and/or processes**
**Other non-ERIC strategies in this category**	
Adaptation of existing processes	Adapted existing MedRec program to fit new intervention or adapt existing technology to meet project needs including health electronic record systems and bed boards. Adapted existing high-risk patient criteria to what MARQUIS2 Toolkit provided. Adapted existing process of medication histories within 24 hours to focusing on patients who will be admitted from the ED.
Alignment of the project with existing initiatives	Aligned project with the quality improvement infrastructure of the hospital and with existing infrastructures like committees (medication safety committee, patient safety committee) that are multidisciplinary in nature. Used existing pre-surgical phone call to obtain a medication history during surgery prep phone calls.
**Attend to professional roles (non-ERIC category)**	**Strategies that promoted implementation of team members and professionals who used the MARQUIS Toolkit intervention feeling valued and having clarity about their roles in the implementation of the toolkit**
**Other non-ERIC strategies in this category**	
Professional roles and task responsibilities.	Specified team member roles and share with key stakeholders, clarify and specify the role of the implementation team in the context of the project within the organization. Frequency of team meetings and assignment of roles and responsibilities to each team member.

*Note*. *Represent sub-categories within main categories as determined by the ERIC taxonomy

*ERIC* Expert Recommendations for Implementing Change, *MedRec* Medication Reconciliation, *EHR* Electronic Health Record, *ED* emergency department

#### Restructuring

Restructuring strategies that altered staffing, professional roles, physical structures, equipment, and data systems were used to implement the toolkit (see Table 2). As one hospital’s team member noted: “We just restructured our current workflow, and we actually had [pharmacist] come on as a new hire. . . made sure that she [pharmacist] would have some dedicated time to do this [MedRec].” Several implementation strategies were used, with changing record systems and changing workflow systems reported across all sites and mentioned extensively by participants. For example, hospitals *revised professional roles* by allowing non-physician professionals to document medication-related changes in the electronic health record (EHR) or health information technology (HIT) systems and shifted the MedRec responsibility to pharmacy technicians (Pharm Techs) or reallocated MedRec tasks from the physician to nurse practitioners. Hospitals changed record systems by adopting and/or adapting electronic processes to better support MedRec, including the use of tracking boards and specific icons to identify patients with priority need for MedRec.

Keyboard shortcuts with MedRec-related common phrases and special medical software programs enhanced documentation and communication between disciplines. Workflow system changes included the addition of PharmTechs to MedRec workflow, their oversight by pharmacists, and a new EHR progress note to facilitate PharmTechs documentation in the EHR.

#### Quality management (QM)

Participants reported several QM steps including the use of data and reporting for monitoring and evaluation. As one participant noted: “With the QI team we did process mapping and then we did an HFMEA [healthcare failure mode and effect analysis] and then we just picked through the various problems and went after them.” Quality management strategies mentioned by most participants across all sites include the use of advisory boards and audit and feedback. Advisory boards included physicians, nurses, pharmacists, patient safety officers, quality improvement staff, and patient safety managers. They typically facilitated connecting various departments to help improve workflow efficiency and to remove implementation barriers.

Audit and feedback included providing leaders with medication discrepancy rates and providers with gaps in individual performance such as report cards on the accuracy of PharmTechs medication histories. Feedback occurred on a one-on-one basis or at meetings. Pharmacists used a mobile application to observe and provide feedback to the PharmTechs MedRec related activities and behaviors. Purposefully re-examining implementation strategies was another strategy used to reevaluate how the hospital’s participation in MARQUIS2 was proceeding. Most hospitals used change management processes to guide implementation, like telling people why MARQUIS2 participation was important and providing evidence of medication errors avoided to demonstrate the value of MARQUIS2 participation.

MARQUIS mentors, as *quality improvement advisors*, in addition to local hospital quality experts (e.g., nursing quality management, performance management/improvement, and director of quality improvement), played a key role in implementation. Tools developed for *quality monitoring* included, for example, Excel spreadsheets to track the number of patients seen by pharmacy technicians, an EHR template to assess MedRec quality, and customizing EHR reports for quality monitoring. Implementation teams obtained *consumer feedback* from physicians, nurses, and pharmacy staff (consumers of MedRec) on the MedRec changes they wanted to see implemented, the efficacy of MedRec notes in the EHR, and the helpfulness of EHR pharmacy notes during care delivery. Feedback resulted in improvements in EHR processes including adaptations to existing EHR MedRec modules and the addition of EHR templates for interdisciplinary communication.

One implementation strategy not described in the ERIC taxonomy emerged from these data. This strategy differs from audit and feedback as it focused on developing specific structures and processes to facilitate monitoring individual performance. This strategy, labeled as “determining project-related goals for individual performance measures,” centered on activities for monitoring individual’s performance as they related to the project’s resource needs and overall goals. Included here were monitoring an individual’s time to perform MedRec, the number of MedRec cases completed per hour, and developing a rubric of expectations for MedRec and using the rubric to evaluate staff performance of the MedRec PharmTechs.

#### Planning

Implementation teams prepared for implementing the toolkit in many ways. For example, one participant noted gathering information “. . . we actually did a survey, a baseline survey to providers, and . . . we also added in our own questions to really sort of help light the fire in terms of, "Do you think this [MedRec] is a good process or bad process?” They also garnered stakeholder buy-in by marketing MARQUIS2 as “This is our ticket, this is our way to get things fast tracked, where we could get experts to support us.” Implementation teams *conducted local needs assessments and assessed for readiness and identified barriers* using Gantt charts, swim lane evaluations, and gap analyses. They built *stakeholder buy-in* through strategies like identifying and preparing champions who would engage staff in MedRec and help to garner support from key stakeholders. Participants involved *executive boards* by reviewing with them MedRec data, reporting on how the MARQUIS project was going, and asking for their help with the selection of units for participation.

In addition, hospitals focused on tailoring their implementation strategies to *overcome organizational barriers and honor preferences* by determining what was achievable for the hospital. For example, some hospitals focused on improvement of MedRec at discharge or overcoming communication challenges by setting clear expectations for physician communication with pharmacists regarding patients discharge medication lists. In addition, *relationships were developed* by building a coalition such as a MedRec steering committee or implementation team.

#### Educating

Education was provided to inform stakeholders about the toolkit and associated implementation efforts. As participants noted “. . . staff meeting . . . we did a kickoff to explain it [MARQUIS2] and did a little presentation about it.” and “. . . we did some presentations at our physician meeting. . . at the patient safety meeting.” Most participants and research sites reported the strategies of conducting educational meetings and informing local opinion leaders. Educational materials included pocket cards, help sheets, electronic modules, and checklists to educate staff. *Ongoing consultation* with quality improvement experts, pharmacy staff, and the MARQUIS mentor, and, *conducting ongoing education* of new and existing staff occurred throughout the year or when performance gaps were identified. Educational meetings were conducted with different professional groups (e.g., hospitalists) and key stakeholders. Training sessions were individualized to meet the needs of each profession (e.g., PharmTechs versus physicians). To *inform and influence stakeholders*, information about MARQUIS2 was spread to the community using mass media (e.g., TV presentations and newspaper advertisements) and posters and emails to inform hospital staff.

### Minor themes of strategies important for MedRec implementation

Two other ERIC categories, finance and attend to policy context, emerged from the data. However, participants mentioned these implementation strategies less often, so they were classified as minor themes of strategies.

The use of incentives and reallocation of funding was important. One participant noted “... our institutional goals to have BPMH completed for our three intervention units. We met that goal and so all of the staff that were involved in meeting that goal got a $25 gift certificate or gift card.” Another noted “. . . but it’s a pay-for-performance incentive for our providers. . . [them] documenting a good MedRec on admission and discharge. . . also the actual discrepancy rate.” Participants reported highlighting instances where the use of the toolkit mitigated risk and reduced liabilities or prevented adverse drug events by identifying and correcting medication discrepancies. Participants rarely mentioned the ERIC finance strategies of *accessing new funding*. Very few hospitals accessed new funding through other grant funding mechanisms (other than the AHRQ funded MARQUIS grant) or allocated risk pool funding that remained at year-end for use to foster MARQUIS2 implementation.

Participants rarely mentioned the use of attend to policy context strategies, even though MedRec is a Joint Commission National Patient Safety Goal (NPSG.03.06.01, https://www.jointcommission.org). Participants at only one hospital noted an upcoming accreditation hospital visit as a driving force for MedRec changes: “. . . their next round of accreditation, and if we don’t have a process in place, then they’re going to have a big problem. That is extremely helpful, that accreditation. I’ve been trying to work on medication reconciliation for probably 10 years. . . but really didn’t start to gain any momentum until. . . our last accreditation cycle”. At this hospital, organizational policies were changed to improve the robustness of their MedRec program by allowing non-physician clinicians to correct medication lists in the EHR, which improved the MedRec workflow in the hospital.

### New themes of implementation strategies not captured by the ERIC taxonomy

#### Integration

Integration of the MedRec interventions was possible by using the strategies of “Adapting existing interventions” and “aligning with existing interventions”. Hospitals used integration in different ways. As one participant noted: “Ever since we enrolled in MARQUIS. . .there were some tools in place already, but I think we adjusted them and changed them, based on the findings of MARQUIS1 to make sure that we followed the MARQUIS2 protocol.”

*Adapting existing interventions* included adapting high-risk patient criteria provided in the toolkit and the medication history module in the EHR. Participants reported A*lignment with existing initiatives* occurred by using the existing pre-surgical phone call to obtain medication histories and aligning the MARQUIS project with the hospital’s strategic quality plan, which helped with garnering support and resources for the project.

#### Professional roles and task responsibilities

Many participants felt the need for clear direction on the *roles and task responsibilities* of the various team members, given that MedRec requires collaboration across multiple disciplines. One participant noted their struggle “. . . who was going to be responsible for taking the best possible medication history at the time of admission . . . Eventually we agreed that it would be the sole responsibility of the pharmacy technician and no longer be a requirement for the nurses or the MDs.” In addition, participants felt that PharmTechs were empowered to play a significant role in MedRec by developing EHR templates and receiving access to areas of the EHR not previously available to them to foster effective and independent work.

### Recommendations for future success

At the end of the interview, we asked participants “Now that you have done it, what would you do differently carrying out the QI initiative?” Dominant themes focused on the project and people. **Project** recommendations included increasing the scope of the project, either through more diverse interventions, disseminating it to other hospital units, or making their MedRec programs more robust: “So I would increase the program, for them to do reconciliation on patients not admitted through the ED.” Recommendations regarding people included examining who the right people are for the team. “I think before even embarking on another journey like this again it would be, do we have the right people and what is it going to take to get there”. The need for project consultation and planning early on with stakeholders were noted. “I think what we could do differently is maybe arrange a meeting with all the leaders across the hospital . . . so if we could get them to come to a meeting, like maybe nursing, and the emergency department, at the very beginning of the project, to kind of share our ideas, and brain storm what we could do together to work in collaboration. . . letting everyone know ahead of time what we are doing with the project.”

## Discussion

Members of MARQUIS2 hospitals reported using a variety of implementation strategies during MARQUIS Toolkit implementation, 32 of which aligned with the ERIC taxonomy and the plan, educate, restructure, and quality management categories (Table 2). Strategies related to finance and attend to policy were rarely cited. Importantly, this study identified two additional categories—integration and professional roles and responsibilities—and 11 new strategies not previously described in the ERIC framework. Our findings suggest that complex interventions like MedRec can use the ERIC taxonomy. But additional descriptors are needed to capture the diversity of approaches used to address hospital contexts such as the availability of resources (e.g., ED based pharmacy technicians, decentralized pharmacists), existing structures (e.g., quality improvement departments; EHR MedRec modules), and processes (e.g., physicians consulting with pharmacy prior to writing the discharge medication list).

Powell and colleagues [30] reported examples of how hospitals could operationalize the various strategies (see Additional file 6 in their article). This research enhances and refines their work by demonstrating numerous ways through which hospitals executed various strategies in practice (see Table 2). For example, for audit and feedback, both Powell et al. and this study identified the use of a variety of sources like medical records and direct observation to obtain information. Similarly, hospitals translated into practice the strategy called “change record systems” by modifying progress notes and treatment plans to reflect the implementation of evidence-based practice, in this case the MARQUIS Toolkit.

This study is the first study to use the complete ERIC taxonomy to investigate implementation of MedRec, a complex interprofessional healthcare intervention. The study findings provide hospitals who wish to adopt MedRec interventions with evidence regarding the most employed strategies from the ERIC taxonomy as well as several novel strategies and specific tactics to execute selected strategies in real-world settings. Our study aligns well with others that have successfully used the ERIC taxonomy of implementation strategies to investigate the implementation of different healthcare interventions (e.g., hepatitis virus C treatment) [38]. These studies have similarly found that not all strategies were applicable to their setting or intervention during implementation [39, 40]. Specifically, the most frequently used strategies included data warehousing techniques (e.g., dashboards) and intervening with patients to promote uptake and adherence. In contrast, in the current study, most sites used predominantly planning and education-related implementation strategies (Table 2). This further shows the need for research on using ERIC for a variety of interventions.

Other MedRec studies have demonstrated the need for implementation strategies including hiring pharmacists, adopting an electronic MedRec system [28, 41, 42], and using multidisciplinary groups to plan for implementation [43]. In addition, prior studies have identified the need for planning strategies [19] such as the use of executive boards and/or sponsors like medication safety boards [44] to enhance the implementation of medication safety strategies. MacKeigen et al. [45] examined the implementation of a reimbursed medication review program and found important strategies to include task shifting, staff training, and software adaptations. Researchers have also emphasized the need for implementation strategies such as ongoing education [46] standardization such as scripts to assist physician-patient discussions and the use of multidisciplinary teams [47, 48], role clarification [49], and audit and feedback [48]. However, this study goes beyond prior research by using the holistic, theory-driven ERIC taxonomy. In doing so, the current study integrates and summarizes previously reported strategies into a cohesive, conceptually organized approach to guide the future implementation of MedRec interventions.

Our work demonstrates the importance of multimodal and multifaceted implementation strategies for implementing complex healthcare interventions like MedRec. This finding aligns with others who have similarly found that implementation strategies were multifaceted [45]. The MARQUIS Toolkit challenges implementation efforts because it is comprehensive consisting of multiple components and organizational levels (i.e., organization, unit, individual staff member or patient) to change entrenched behaviors [50]. Thus, implementation strategies and tactics must target behaviors and the different organizational levels to facilitate change and improve organizational performance, and this is only possible when employing a multifaceted approach to implementation.

Increasingly, researchers recognize the need to tailor implementation strategies to specific contexts to overcome their specific barriers and make use of their unique facilitators [51, 52]. Future research should build on this study’s findings regarding implementation strategies and tactics to examine the contextual antecedents (e.g., leadership, team cohesion) that may drive the selection of particular implementation strategies and approaches to implementing them. It would be especially valuable in the context of MedRec to investigate the contextual factors that drive the selection of strategies, the association between implementation strategies (and operationalization of them), and clinical outcomes like unintentional medication discrepancies as well as, for example, implementation outcomes such as feasibility, acceptability, fidelity, and sustainability [53] of MedRec interventions in hospitals that participated in the study.

This study was limited to MARQUIS2 participating hospitals selected in an application process that required executive leadership support and a desire to improve their MedRec processes. Thus, participating hospitals were contextually at a high level of readiness, without the need for new policy-driven strategies. It is possible that our findings would be different in hospitals less ready to change. For example, although The Joint Commission lists MedRec as a national patient safety goal (NPSG.03.06.01), accreditation was not a major driver for MARQUIS2 participation but rather gap analyses of existing MedRec processes and other motivators, such as reduced staffing levels in the emergency room of nurses who completed MedRec. Accreditation bodies can play a significant role in forcing change in organizations resistant to change but their role in organizations with a high level of readiness appears muted. Although this study did not find finance and policy context to be common drivers, the researchers still recommend their inclusion in future efforts involving more uncertain policy contexts and with hospitals where readiness for change and contextual factors driving implementation might be different.

We identified two additional categories of the Integration and Professional Roles and Responsibilities. Integration through adapting existing structures and processes and aligning the project with existing initiatives likely enhanced implementation. Integration of newly adopted interventions is key in quality improvement and system change [54, 55]. Given the vital role of adaptation to the sustainability of interventions [56, 57], using these integration strategies in the implementation process will likely also enhance sustainability. Future research should further explore the strategies most likely to facilitate implementation sustainability. Regarding professional roles, specifying the role of team members in implementation efforts reduces role ambiguity, which plays a role in employees job stress, satisfaction, overall performance, and employee turnover [58]. Role clarity also facilitates interprofessional collaboration [59], which is vital for success of interprofessional implementation teams. Clarifying roles might also limit the duplication of team members’ efforts and thereby ensure the judicious use of organizational resources. Given the interprofessional nature of MedRec and many healthcare interventions, role clarification might be a particularly important strategy.

### Study limitations

Although the study sample size was relatively small as only 16 hospitals participated in this study, data saturation was achieved with consistent themes emerging across hospitals prior to the completion of all interviews. Of the two hospitals not represented in this report, one also demonstrated limited engagement with and did not collect sufficient data during MARQUIS2 study to be included in the analysis of the primary outcomes. The other hospital participated in the larger MARQUIS2 study, but the researchers were unable to recruit implementation team members for interviews. As noted above, selection bias is possible as MARQUIS2 hospitals were limited to those that applied to participate and they may inherently be different from those hospitals that did not apply to participate in MARQUIS2. Although the study results underrepresent the voices of executive leaders, implementation team members indicated ways executives supported toolkit implementation as reflected in the planning strategy of “Involve executive boards and/or sponsors.”

Response bias, including social desirability, are additional potential study limitations. However, phrasing questions to prevent leading participants towards a specific answer, asking questions in a non-threatening, neutral manner, and using simple, unbiased language helped to mitigate this risk. Although generalizability may be limited, the hospitals were heterogeneous in type, size, and location, and thus, the findings may be applicable to hospitals with similar characteristics.

This study used web conferencing to conduct the interviews. Interviews were conducted ethically and confidentially by informing participants of all present in the room like the PI conducting the interview and the RA taking notes and that interviews were being recorded. The use of technology allowed interacting with participants remotely and eliminated the travel time and costs associated with face-to-face interviews. It was the most practical and feasible option to achieve the research aims in the presence of cost constraints.

## Conclusions

This study demonstrated that implementation strategies and how they are operationalized can guide the implementation of complex interventions like MedRec in acute care hospitals. The ERIC taxonomy categories of plan, educate, restructure, and quality management emerged as especially central to implementation efforts. In addition, two novel categories emerged emphasizing the importance of integration and professional roles and responsibilities. The findings of this study also suggest that finance and policy strategies were less important, at least at hospitals ready for implementation. In sum, the ERIC taxonomy of implementation strategies provides a strong foundation for evaluating implementation of a complex healthcare intervention such as MedRec. However, additional categories and strategies were needed to describe the approaches reported. It is likely that the type of intervention or the implementation context might drive the selection of strategies. Future research should explore the drivers of implementation strategy selection and their operationalization. Identifying the most effective implementation strategies to achieve positive clinical and implementation outcomes, which might depend on the hospital context, is also needed.

## Supplementary information

**Additional file 1.** Implementation strategies and supporting quotes

## Data Availability

The dataset supporting the conclusions of this article will not be publicly available due to ongoing data analyses related to all study aims but are available from the corresponding author on reasonable written request.
