# Supplementary material for: Implementation strategies in the context of medication reconciliation: a qualitative study
Source: Implement Sci Commun. 2021 Jun 10;2:63. doi: 10.1186/s43058-021-00162-5 (PMC8193884; doi:10.1186/s43058-021-00162-5)
Supplement: Supplementary file 1 — Additional file 1. Implementation strategies and supporting quotes [file 43058_2021_162_MOESM1_ESM.docx]

| **Additional File 1. Implementation strategies and supporting quotes.** | |
| --- | --- |
| **Planning** | **Representative Quotes** |
| **Strategy: Gathering information** |  |
| Conduct a local needs assessment | *“We did a needs assessment first off to ensure that all stakeholders would have buy-in. When we did the needs assessment we realized that we did have an opportunity, and it was actually something that our medical staff and I had been working on prior to, and I think it was just great timing that we had an opportunity to join the MARQUIS2. Then when we were selected, it was a great match for us.” (White-SL)* |
| Assess for readiness and identify barriers | *“So I think the first thing was, is evaluate our capability of initiating the intervention. But as many institutions, we're short on resources and that's really the first thing that came to mind is who would we have work on this project and how could we reallocate our staffing to meet the requirements of the program.” (Purple-SL)* |
| Identify resources | *“Once we got approval ... So first there was getting internal approval [hospital] but once we found out the external approval [MARQUIS Selection] it was kind of just, go for it. So for us it was beg, borrowing and stealing and liberating staff everywhere we could to make it happen.”(Navy-FG)* |
| **Strategy: Stakeholder buy-in** |  |
| Identify and prepare champions | *“That was headed up by our Director of Quality and Patient Safety because she was appointed as the lead. I'm not sure exactly, I don't exactly know how our physician champion was selected. I think it had come through our Chief Safety Officer, and then he selected medicine units as the primary areas that he had the medical director of the medicine unit. From there, when we had a scope, our quality and safety person pulled in people from different groups like medication safety, nursing practice.”(Green-TM)* |
| Involve executive boards and/or sponsors | *“Then in addition to just the numbers, we were reviewing medication events such as delay in care, wrong drug, wrong route, so it wasn't as streamlined as what the MARQUIS2 data showed. It was more so just a snapshot of one-off incidents, physicians bringing up incidents that they've encountered or were just reports of pharmacists finding incidents.” (White-SL)* |
| Recruit, designate, and train staff | *“Yeah. We hired several new technicians and we trained them, both didactic training session, a three hour didactic class, teaching them about MARQUIS and then also the hands on training afterwards.”(Grey-FG)* |
| Conduct local consensus discussions | *“We set up times for feedback to that group, and that was basically it. We just prepared them with the details of the program, talked about how long we would be participating, what kind of deliverables we thought we would get from the grant, and how we would apply those in the organization.” (Maroon-SL)* |
| Marketing to stakeholders | *“But, we essentially marketed MARQUIS2 as, "This is our ticket, this is our way to get things fast tracked, where we could get experts to support us", and then we'd be one of the first hospitals to get ... You know, we're very competitive, and so we also twisted it into a competitive thing, where we could be part of a publication, part of the national leaders, and something we care to adopt.”* (White-SL) |
| **Strategy: Select an implementation strategy** |  |
| Tailor strategies to overcome barriers and honor preferences | *“Part of our other barriers were proper identification and proper prioritization, and with the teamwork of medical staff, with our IT System, with nursing, we were able to streamline that to know pinpoint identification of admitted patients into the process where we have today, where on our Cerner tracking board, need to admit patients are now flagged as an icon.  MedRec techs see that, they go to those patients. They are queued up in their queue of priorities, and then they call the physician saying, "Administrator done, you're now able to continue the admission process." (White-SL)* |
| Stage implementation scale up | *“Once we were able to document that was successful, that it brought value to the care we were giving to the patient in an accurate medication reconciliation history, they endorsed the program to run full-time with a full complement of FTEs.” (Maroon-SL)* |
| **Sub-theme: Develop relationships** |  |
| Build a coalition | *“Or that we had a team that represented the problems from 360 degrees is really important. And we did that both by open-invitation and then I would email leadership to get recommendations for people. So I emailed nursing leadership saying that we were doing this project and we were looking for representatives.” (Gold-FG)* |
| Partner with Quality Improvement experts external to the organization | *“I think the MARQUIS2 team did an excellent job of guiding the data collection process, creating the random number tables, and various other things like that. It was a matter of getting people, taking a process that had historically been done on paper by our pharmacy team and converting it over to REDCap with a few other enhancements per the MARQUIS protocol. It took some transition but I felt like it was facilitated very well by the MARQUIS2 team.” (Magenta-SL)* |
| **Other non-ERIC strategies** |  |
| Develop interdisciplinary implementation teams | *“So we had the clinical coordinator, the director of pharmacy, myself, some pharmacy residents, and we also included one of the hospitalist groups. We have 2 hospitalist groups, we included a representative from one of those hospitalist groups.”(Purple-SL)* |
| Identify people and time necessary for data collection to support the project. | *“Well, I would say that we underestimated the amount of work that it would take and the time that it would take for our staff to do the data collection and submit it all, so if I was going to do it again I would try to get a better sense of how long it would take to collect data on each patient and then make it very clear to the people that we're going to do the work exactly how long it would take so they could dedicate time and schedule it off, sort of monthly. Because one of the things that did happen is that because we under appreciated the amount of time it would take, they got behind and then they were kind of playing catch up. And it caused a lot of anxiety for us as a team. They were often, it felt like were scrambling at the end of the month to catch up with the data.”(Grey-FG)* |
| **EDUCATE** |  |
| **Strategy: Develop materials** |  |
| Develop effective educational materials | *“We created a lot of educational tools. Then we had an internet site with the MedRec resources posted for the organization, because here at (Hospital), it was a shared responsibility among staff in gathering that medication history. A lot of the resources to support all members are posted on that site. Then we also got support to hire one technician at each of the emergency sites. We have three larger sites. . . We had technicians at each of those sites, got support for them, but what I created was a pharmacy or technician certification program and they underwent how to take a medication history accurately.” (Pink-FG)* |
| **Strategy: Educate** |  |
| Provide ongoing consultation | *“So we had done that, we prepared a document, we provided in person education, we made ourselves available basically for anybody who was working in that capacity, but have not seen that function before, we would make ourselves available if they had any questions, and give them a demo, and evidently if someone doesn't work in that shift very often, they may not have seen it for some time, so they are always welcomed to ask more questions, based on that.”(Silver-TM2)* |
| Conduct educational meetings | *“We had a face-to-face meeting where we talked about the study and were pretty specific about what the commitment would be, how long it would be, how many hours a week it would be, the importance of the study. I think that was the other sort of big thing was really emphasizing. The need and the opportunity I think, to be a kind of pioneer site.”(Gold-FG)* |
| Make training dynamic | ***“****She's* [pharmacist] *the one that created the staff training. She ran it by myself and one of our pharmacists. Essentially it was a checklist, and it was essentially, we showed the technicians how to go through it, had to go through the process from beginning to end. Then when you shadowed the tech, sorry it's a term for technician, we then shadowed the technician to ensure that they were asking the right questions, and putting the data correctly into the charts. Then we transitioned from shadowing to, "Alright, you're on your own",  and then (XXXX) would go back and spot check 10 or 20 records to confirm that the histories were done correctly, then we would learn some type of feedback in regards to that.“(White-SL)* |
| Conduct educational outreach visits | *“. . . there was supposed to be a training class in [X] and we were going to send a couple people, and then we connived and got our way, and instead, the training class came to our hospital instead. We offered to pay for the food . . . and we actually were able to get over twenty-five associates from our hospital at the Med Rec class. So for us, that was a win-win because everyone got to hear about all the risks of not doing a proper Med Rec, but then also got to plan on how to do a Med Rec and role play, which was wonderful, so that was very helpful.”(Navy-FG)* |
| Conduct ongoing training | *“The biggest thing was to be able to continually assess... We needed to keep giving them appropriate education to be able to make sure they were able to do what they needed to do, so a lot of feedback of data, a lot of discussing about where they had gaps in their performance, that kind of thing.”(Maroon-SL)* |
| Distribute educational materials | *“the weekly emails, and the monthly meetings as well. The biggest thing from the pharmacy side was just kind of be available for any question that came up because it was new for a lot of people and they needed a lot of coaching in the beginning to understand it. But just kind of reiterating, we even made a little laminated guide for them and put it in the pharmacy, so that they can refer to it, how to do the med rec step by step. So we try to make resources available, whether it's people or some sort of published guides for them to look at.”(Silver-TM2)* |
| **Strategy: Educate through peers** |  |
| Inform local opinion leaders | ***“****Yeah so, I think, again, our clinical leaders along with myself and our CMO (XXXX) take opportunities to educate and bring this to people's attention at various committees as well as leadership meetings. There are both formal and informal ways to advocate for a program and we tried to use all of them. Ultimately, getting support for resources is always a bottom line issue.”(Orange-Lead)* |
| Create or participate a learning collaborative | *“ So, part of it was tying it to the overall medication history and reconciliation project that (XXXX) had mentioned. We got this business plan. We were able to justify that and to get us to be able to do MARQUIS, part of it was to be able to say and show that there is some data that shows when you participate in a collaborative that you're more likely to be successful with implementation of a new program, and so that was part of the rationale for doing it and the justification of why to participate. And I would say that we've seen some those sort of benefits from that because we're able to ask questions to different people across the country. It also provided some technology and infrastructure around our quality process that we would have been hard-pressed to put together on our own and it also gave us sort an outside set of eyes to look at our process that kind of validated the quality of the work that we're doing.”(Grey-FG)* |
| Provide ongoing consultation | *“So we had done that, we prepared a document, we provided in person education, we made ourselves available basically for anybody who was working in that capacity, but have not seen that function before, we would make ourselves available if they had any questions, and give them a demonstration, and evidently if someone doesn't work in that shift very often, they may not have seen it for some time, so they are always welcomed to ask more questions, based on that.”(Silver-TM2)* |
| **Strategy: Inform and influence stakeholders** |  |
| Use mass media | *“Last year, we had a little TV blurb and then a newspaper article. We did a lot of education with our patients out in the community. Then just with community partners, we've had a couple engagement sessions with them learning on some of the challenges that they're faced with items that they receive from us, so we have this open transparent communication between the community and us, and we just present the successes and challenges at the meetings. . . We also look at patient education, so there was a big push for education in the community. We do have marketing out there with the social media.”(Pink-FG)* |
| **Other non-ERIC strategies** |  |
| Individualized training sessions | *“Then that would help drive some of the numbers, but then it came out to be, "Well, I've [*physician] *noticed that in* [EMR system] *, I'm doing what I'm supposed to, but I'm not getting credit, why?" Then we* [pharmacy staff] *would do some more investigations, and say, "Okay, we have to refine the* [EMR system] *tool", or "I* [physician] *did the MedRec, but why is my quality poor?" Then we* [pharmacy staff] *go back and was like, "Actually, you did the MedRec before the history technician was even able to imprint it, so you actually did a MedRec of a prior history that wasn't then truly done by the technician". Then we had to walk through the process saying, "This is how you know when a technician has done it versus something that* [EMR system] *had pooled from years ago." (White-SL)* |
| **FINANCE** |  |
| **Other non-ERIC strategies** |  |
| Incentivize positive performance and training | *“I think what worked well was, from my perspective was the hospitalists getting them onboard, was having business meetings where we would discuss issues, giving them Starbucks cards for BPMH training . . . and that got them engaged into ... which can sometimes be dry material.”(Orange-FG)* |
| Demonstrate value to justify program and gain ongoing support | *“Positions, I think, in health systems always have to be justified and what not, at least in our health systems on the (Place), it's just been a struggle through with the Health Care Reformation, so we've had to constantly collect data through our reviews and the value that the program brings.”(White-SL)* |
| **RESTRUCTURING** |  |
| Revise professional roles | *“We also had a pharmacy technician that was helping in our surgical admission center which is a group of nurses that call all patients that are scheduled to an elective surgery and she was working with one service line, to help complete BPMH for patients that were being admitted for an elective surgery and so worked with that group to try and train them on BPMH.”(Orange-FG)* |
| Change records systems | *“ We also had what the MARQUIS mentors coined one of our IT systems as the measurevention. We had this system, where one of our IT representatives created which allowed for two separate electronic systems to talk to one another, and identify who in the hospital has a BPMH and who still requires a BMPH to be completed.”(Pink-FG)* |
| **Other non-ERIC strategies** |  |
| Facilitate relay of clinical data to inter-professional teams | *“We realized that our biggest barriers were in regards to documentation of our results, making sure that it was fully disclosed, transparent to obviously the provider that needed information. So, we had to get through barriers in regards to allowing pharmacy technicians to actually input the data that was required in the physician's field, and not a technician window that no one sees.”(White-SL)* |
| Change workflow systems | *“The pharmacists would do the review and reconciliation in [and] document and then note any changes and then prior to MARQUIS we had a separate transitions of care consult order that they could place and that would be for discharge reconciliation bedside counseling and bedside delivery of discharge medicine and so we eliminated both of those consults and merged the group so that the pharmacy team, when they were doing MARQUIS it was kind of ... we'd like a patient to get all of the components. So patients that had a BPMH on admission would get the discharge reconciliation and discharge counseling. If they didn't have a BPMH on admission, we tended not to do discharge reconciliation or discharge counseling, we didn't really know what patients, we couldn't confirm what patients were actually on.”(Orange-FG)* |
| **QUALITY MANAGEMENT** |  |
| Use advisory boards and workgroups | *“We have our own pharmacy specific quality improvement group and the MHDs have always, or at least for a very long time, even before MARQUIS, They were embedded in that part of the department, so we kind of naturally started with them.”(Grey-FG)* |
| Audit and provide feedback | “*The biggest thing was to be able to continually assess- to provide them [med techs] with appropriate ... We needed to keep giving them appropriate education to be able to make sure they were able to do what they needed to do, so a lot of feedback of data, a lot of discussing about where they had gaps in their performance, that kind of thing.”*(Maroon-SL) |
| Purposefully re-examine implementation | *“That sounded like the right place* [Emergency Department] *to do it* [MedRec] *but reality of the chaos in the ED and the lack of privacy there and the problems with guessing whose gonna be admitted to what ward and all those sorts of things led to some re-thinking about that* [having pharmacy technicians in the ED] *and now the pharmacy techs are* [instead] *actually embedded on the units close to the ED.”(Orange-Lead)* |
| Use a quality improvement / implementation advisor | ***“****Yeah, and I think recently within the last six months we partnered with our performance excellence groups and I think that that's really helped get the information out in a way that the higher ups and the seasoned people will understand. They've helped translate it into what they care about and we presented in a couple of meetings and I think looping a group like that in earlier may have ... I mean it's hard to say because we can't go back in time, but it may have helped, I think so that we could be where we are now six months ago so that if there were additional things that we wanted to do while the MARQUIS study was still ongoing, we would have had the time and support of the institution to make those changes while the study was still ongoing theoretically. . .”“(Orange-FG)* |
| Develop tools for quality monitoring | *“We had then other staff members, another aspect of one of the things that we implemented was standardized process and standardized documentation. We did the training, we implemented the standardized note and standardized documentation. Then we actually recreated a rubric. We randomly pulled some ... for each staff member we would pull a few of their notes, review it against the rubric and provide them feedback. But we also used that in their annual evals to see how well did they follow the standard and implement this in their areas.”(Green-TM)* |
| Determine project-related goals for individual performance measures | *“ So, for one is that as part of their performance we have a score card of activities that they're expected to complete. Then the pharmacist reconciling the medications after they're ordered and after the history is taken by the technician, that's one of their requirements and they do have a merit based performance review. So, that is definitely incorporated” (Grey-FG)* |
| Obtain and use consumer and family feedback | *“Really involving them early on to make the decisions on how to make the change. Then we utilized those individuals as our champions as well as got their feedback on a regular basis. As managers, we tried to touch base with all of our staff and see how things are going and provide the feedback and check in, but it was also nice to have those day to day frontline pharmacists and pharmacy technicians frequently touch base with us and say how are your colleagues doing? What are you hearing? What are the barriers now that you've used this in this system for a while, how is it going?” (Green-TM)* |
| Determine ownership and hold individuals accountable | *“From our department was we have really taken this as an ownership. I think continuing to display the ownership of it by having it displayed as an organizational goal, by setting it as shared goals amongst teams.”(Green-TM)*  “*So I think that, as we're trying to define the different responsibilities and to decrease how many errors we have and delegate different components of the matter of process to different people, we have to somehow . . . make them accountable for the different processes. . . . And at the heart of it, and this is something that we also try to focus on, is the patient. So, educating the patient of their own responsibility, or the caregiver, in this process.”(Silver-TM)* |
| **POLICY CONTEXT** |  |
| Use accreditation bodies and organizational policies to direct change | *“Based on our Accreditation [body], there are certain organizational requirements, and so I would look and audit those pieces for positions nursing and pharmacy to see if they complete the forms based on our standards. I would audit them for that, and then provide feedback as just little reminders and tips for next time, and how to ensure that they complete it, and complete it with accuracy. . . .* *Part of that is that we had a burning platform in [confidential] that Accreditation [body] requires all hospitals to have a process for medication reconciliation on admission, transfer and discharge . . . .”(Pink-FG)* |
| **PROFESSIONAL ROLES AND RESPONSIBILITIES**  **(Non-ERIC Category)** |  |
| Implementation team member role specification | *“I think that what requires success is just making sure that people are aligned, they understand their role, they understand the mission. Essentially, everyone needs to understand the why, and then we go back to understanding, "Okay, so how do I do this, and what's my role?. . . I think . . . that in my role as the leader of the site, making sure that I always stress communication. I always stress everyone needs to understand what their role is, how they're contributing, celebrate your successes, and then continue to talk about opportunities for improvement.  "(White-SL)* |
| **INTEGRATION**  **(Non-ERIC Category)** |  |
| Adapting existing processes | *“We have made some optimizations, just in general after the electronic medical record that we use has a pretty good medication history module, but we did optimize it to our needs and there were some workload. For instance, patients were scheduled for admission due to a scheduled surgery, elected surgery. We actually had to come up with a new work flow for that and there were some additional ... There were some additional modifications that we had to make but they were I guess on the minor side.”(Grey-FG)* |
| Aligning with existing initiatives | *“I think another thing that really helped us to in terms of the MARQUIS is aligning it with existing initiative that we had in the hospital, the champion care medication reconciliation project was ongoing before the MARQUIS came over. Once MARQUIS came, we just merged it to continue the infrastructure and the support without ... that kind of helped aligning it with initiatives.”(Orange-FG)* |
| *Note. Parenthesis represent a participant of a particular hospital. Colors = hospitals; SL=site leader; TM=team member; FG=focus group. ERIC= Expert Recommendations for Implementing Change; MedRec=Medication Reconciliation; EMR=Electronic Medical Record; ED=Emergency Department* | |
